# Acoustic conditioning of the neonatal incubator compartment: Improvement proposal

**DOI:** 10.3389/fped.2022.955553

**Published:** 2022-09-08

**Authors:** Víctor M. Rodríguez-Montaño, Juan Luis Beira-Jiménez, Virginia Puyana-Romero, José Luis Cueto-Ancela, Ricardo Hernández-Molina, Francisco Fernández-Zacarías

**Affiliations:** ^1^Acoustic Engineering Laboratory, University of Cádiz, Cádiz, Spain; ^2^Acoustic Environments Research Group, Department of Sound and Acoustic Engineering, Universidad de Las Américas, Quito, Ecuador

**Keywords:** neonates, sound pressure, incubators, noise source, NICUs

## Abstract

The objective of this work focuses on the study of the main sources of noise associated with incubators and the acoustic effects that derive from them. The method that has been established is based on tests carried out under different operating conditions of the incubators. Noise levels are analyzed under different boundary conditions (Neonatal ICU and “Controlled environment rooms”). Under normal operating conditions, the levels inside the incubator are around 56 dB (A), values that exceed the maximum limits recommended by the American Academy of Pediatrics. The scope of this study is to evaluate the existing noise levels in the incubator and analyze possible design improvements. The study was carried out in the hospitals of Cádiz, Huelva, and Malaga.

## Introduction

Since the launch of the Neonatal Intense Care Units (NICUs), the growing evolution and incorporation of technologies in the treatment and care of newborns have substantially modified the ecology of these units, transforming them into an environment with high levels of sound pressure, uninterrupted luminosity, and intense work rhythm. This environmental situation, whose perception of the premature neonate was minimized while it was inside the maternal uterus, can significantly compromise the health of patients given their immaturity.

The tests carried out in neonates, using an electroencephalogram of the spontaneous activity induced by the environment to which these patients are subjected in the NICU, reflect tense wakefulness and intensified fatigue (1, 2).

This implies that sleep deprivation affects several physiological parameters, which can negatively influence the recovery of neonatal patients. The cause of this alteration is due to multiple factors including the patient’s medical condition, medication, respiratory care, treatments, care procedures, light, and noise.

However, it must be remembered that unlike the factors related to the condition of the newborn, noise as a source of sleep disturbance can in many cases be avoided. It is estimated that approximately 40% of these disorders are due to noise (3). In this sense, Spanish legislation on occupational risk prevention (4, 5) indicates that being subjected to high levels of sound pressure can generate both physical and mental disorders, in the short, medium, or long term, and may even cause, the worst-case, permanent incapacity of the workforce, regardless of the legislation, the exposure to noise of the patients.

The American Academy of Pediatrics (AAP) has observed that prolonged exposure to sound levels above 90 dB (A) leads to hearing loss among other medical complications, and therefore recommends that noise levels within the NICU should stay below 45 dB (A) (6). For its part, the World Health Organization (WHO) recommends that the newborn should not be exposed to a sound pressure level higher than 40 dB (A) during the day and 35 dB (A) at night. In Spain, the Standards Committee of the Neonatology Society of the Spanish Pediatric Association (AEP) recommends a total background noise level in the NICU of less than 45 dB (A) and not to exceed, temporarily (7) a maximum of 65–70 dB (A).

As an example, during pregnancy, the fetus is in an environment that contains rhythmic, structured, and patterned sounds that come mainly from the mother. The intensity of the sound recorded internally within the amniotic fluid, in tests carried out on sheep, did not exceed 50 dB (A) (8), confirming the validity of the aforementioned recommendations. Although the external sound is also transmitted to the fetus, this environment is capable of regulating and acting as a filter for the stimuli it perceives, mainly at higher frequencies (9).

Despite all the aforementioned recommendations, numerous studies report noise levels in Neonatal Intensive Care Units that exceed these limits by up to 70% of the newborn’s exposure time, with averages ranging between 55 and 89 dB (A) (3, 10–14). The problem is aggravated, even more when maximum values of the sound pressure level appear, mainly due to the alarms of any of the equipment that surrounds the neonates to the sound of the telephone, etc, in which the sound pressure level is Instantaneous (Lpeak) reaches levels that can exceed 144.8 dB (14) a value well above the pain threshold, located at 120 dB.

The interior of the incubator cabin has its level of background noise due to the noise generated by the motor that controls the temperature and humidity inside the incubator and which is fitted inside. Various studies indicate that the engine generates an average of 55–60 dB (A) (11, 15), while the equipment and activity inside the cabin and its surroundings can contribute between 10 and 40 dB (A) more, therefore, these patients are permanently exposed to a noise level ranging between 50 and 90 dB (A) (16). The UNE- EN 60601-2-19: 2009 (17) standard limits the noise level inside the incubators to 60 dB (A), for test conditions (temperature between 36°C and maximum humidity), clearly exceeding the recommendations given by the different organizations (AAP, WHO, AEP, etc.) as mentioned above.

Taking into account that a newborn can spend very long periods in the incubator compartment, the noise dose is a fundamental factor and, therefore, the noise generated by the incubator itself, fundamentally in periods where the environmental noise is low (nocturnal periods), underestimating the dose to which the newborn is subjected. Therefore, our study aims to (i) know the behavior of the incubator derived from the main sources of noise associated with it, and (ii) the acoustic effects that derive from it.

## Methodology

### Measurement instruments

To carry out the measurements have been used several models sonometers: 2270, 2260, and 2250 Brüel & Kjaer, RION series NL-31 and Svantek SVAN 958A, and the sound source Brüel & Kjaer Model 4224 and calibrator Brüel & Kjaer model 4231. Before carrying out the measurements, all the equipment was previously verified and calibrated.

The recorded data were processed using the Brüel & Kjaer Evaluator Type 7820 software, SVAN PC++, and Microsoft Excel.

### Measurement procedure

The procedure used must allow the traceability of the measurements, so it is important to use resources that allow their repeatability. Therefore, (i) the tests are carried out in unoccupied incubators, located in different spaces according to the objectives to be studied; (ii) A pink/white noise source was used to evaluate the attenuation capacity of the incubator; (iii) To carry out the tests, microphones were placed inside and outside the incubator. Data was stored with a 48 kHz sample rate and 24-bit quantization. The data analysis has been carried out in thirds of an octave; (iv) A test protocol was established, aimed at characterizing the incubator without the influence of the external sound environment of the NICU, in the so-called “controlled environment rooms.” In this sense, a series of tests were carried out in the Semi-anechoic Chamber of the ETSIT of the University of Malaga, and in a room provided by the Virgen de la Victoria University Hospital, in Malaga; (v) To know the spectrum of acoustic energy, environmental noise measurements were made in the NICU of the Juan Ramón Jiménez Hospital during a sampling period of 15 days, to know the noise levels in the room environment and the influence inside the incubator cabin; (vi) At the Puerta del Mar University Hospital, in Cádiz, we proceeded to study the possible effects that the incorporation of sound absorbing material inside the cabin would have, as well as to know the sound pressure levels that the incubator motor transmits inside the incubator cabin.

## Results

**FIGURE 1:**
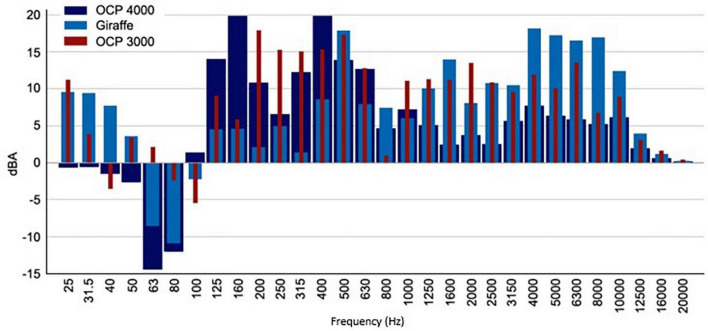
Differences between the sound pressure levels in 1/3 of an octave inside and outside the incubator. Incubators turned on with a sound source emitting pink noise.

Figure 1 shows that the three tested incubators have very different behaviors, in terms of attenuation. It can be observed, in general, that the Ohmeda Medical Giraffe Omnibed (Giraffe) incubator is the one with the worst behavior at low frequencies (between 63 and 1000 Hz). For high frequencies, the worst performing incubator is the Ohmeda Medical Ohio Care Plus 4000 (OCP 4000) incubator, although it also displays it at very low frequencies (below 80 Hz). Halfway there is the Ohmeda Medical Ohio Care Plus 3000 (OCP 3000) incubator, which shows a more regular behavior in broadband and, therefore, among the three incubators, the one that potentially offers the greatest protection to the neonate against noise present in the Intensive Care Unit.

**FIGURE 2:**
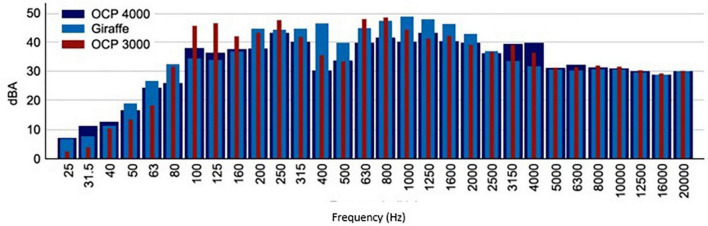
The spectrum of sound pressure levels in 1/3 octave inside the OCP 4000, OCP 3000, and Giraffe incubators were measured in the “Controlled environment room.”

The sound environment inside the cabin generated by the engine is graphed in Figure 2. As can be seen, in all the models of incubators analyzed, the sound pressure level is very high throughout the entire bandwidth, highlighting the range of frequencies between 100 and 4000 Hz, a range of greater hearing sensitivity.

**FIGURE 3:**
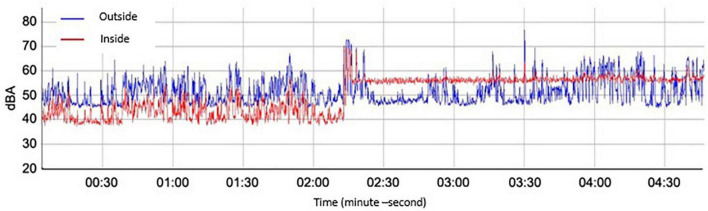
The spectrum of sound pressure levels concerning time was recorded inside and outside the incubator.

If we establish a comparison between the variation of the minimum levels collected by the sound level meter located inside the cabin and outside it, as can be seen in Figure 3 it is verified that the noise levels in the incubator are higher, a fact that can be attributed to the motor located inside to maintain optimal levels of temperature and humidity. Similarly, it is observed that the minimum levels in the incubator are more stable than the minimum levels of ambient noise in the NICU since the noise levels inside the cabin are attenuated by the acoustic insulation it provides. Regarding the maximums between these two points, it can be seen that the maximum peaks present a minimal relationship, associating the values present in the unit with those that the neonate may be perceiving inside the incubator.

**FIGURE 4:**
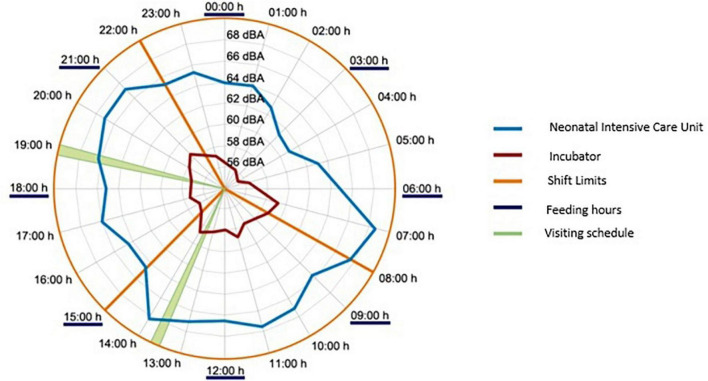
Integrated mean values of equivalent continuous level with 1 h of integration period recorded at the Juan Ramón Jiménez Hospital, in Huelva.

Figure 4 shows the levels of background noise that were recorded in the NICU room of the Juan Ramón Jiménez Hospital in Huelva, given by the 90th percentile (L90), which ranges between 43 dBA, at night, and 62 dBA, in the daytime period (10).

**FIGURE 5:**
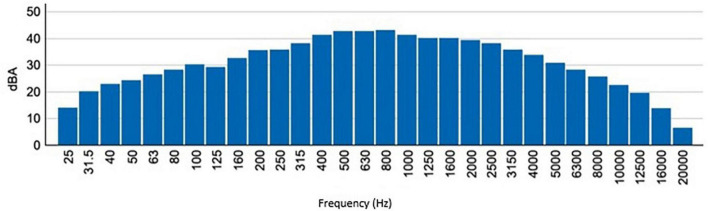
The spectrum of sound pressure levels in 1/3 of an octave recorded in the NICU of the Juan Ramón Jiménez Hospital, in Huelva.

Regarding the noise levels recorded in the NICU, these vary from 42.3 dBA (Lmin) to 97.4 dBA (Lmax), with an equivalent sound pressure level (LAeq) that exceeds 63.7 dBA. The 1/3 Octave sound pressure levels reached in the NICU, and shown in Figure 5, indicate a greater amplitude of low and medium frequencies (<2.5 kHz).

In the tests carried out in one of the rooms of the Puerta de Mar University Hospital, in Cádiz, an attempt was made to evaluate the possible decrease in the sound pressure level when sound-absorbing material in the cabin. Several materials with an absorption coefficient greater than 0.9 were used, such as Rokfont type Teclan 1, Rock wool, and the Wedge type of fiberglass. This material was placed in areas where the newborn’s vision is not necessary (head, foot, and/or dome). The result was not promising, achieving, in the best of the analyzed cases, attenuation of 1.7 dBA.

## Discussion proposals for improvement

Figure 5 confirms the results of various investigations on the environmental noise of NICUs (3, 10–14), the noise levels recorded far exceed the values recommended by the different international entities, such as the AAP or the AEP, recommending that the background noise of NICUs should be kept below 45 dBA (9).

From the measurements made in the “controlled environment rooms,” we can affirm that when the incubators are turned off and the ambient noise levels in the room do not exceed 45 dBA (the limit recommended by international entities), inside the cabin, the sound pressure level is equal to or less than the background noise. When the incubator is turned on, the motor that drives the fan to maintain optimum humidity and temperature conditions inside the cabin causes a background noise level inside the cabin close to 56 dBA and remains constant throughout the measurement period.

For the characterization of the incubator compartment, an external sound source located 2 m from the incubator was used, emitting a sound pressure level of approximately 85 dBA. When we apply pink noise, whose main characteristic is to maintain a flat frequency spectrum in 1/3rd octave band, that is, equal energy throughout the frequency band, both with the incubator off and on, the difference between the two environments is approximately 10 dBA. The attenuation is also maintained when we apply white noise, whose main characteristic is to maintain an increasing spectrum as a function of the frequency in the 1/3 octave band. In short, the walls of the cabin produce insulation of 10 dBA. With these measurements, it was possible to know the attenuation capacity by frequency bands of the walls of the incubator.

With the incubators in normal operating conditions (temperature between 36 and 37°C and relative humidity between 40 and 60%), if the background noise in the care unit is lower than the loudest noise reached by the incubator motor in the interior of the cabin (in this case it would be 56 dBA), situation given at night in the analyzed NICU, the noise dose perceived by the neonate, calculated for a reference period of 8 h [comparison with the dose that a worker will receive during their working hours (5)], will be:

Noise dose = 56 dBA + 10 ⋅ log (t/8)

where “t” is the time that the newborn is in the incubator throughout the day.

Therefore, the dose received by the neonate over 24 h will be increased by 4.8 dBA. Now, if the ambient noise is much higher than 56 dBA (situation simulated by the external sound source), the noise dose received by the newborn inside the incubator room will be less than that perceived by any other individual outside of the incubator. That is, during the daily activity in a NICU the noise is not less than 56 dBA, in this situation the incubator attenuates external noise by up to 10 dBA, but the noise inside the cabin will never be less than 56 dBA (the noise generated by the incubator itself) (15, 18).

With the measurements carried out in the Hospital’s NICU, it was intended to know the sound pressure level that the incubator motor transmits to the interior of the incubator. To do this, following the criteria established in the R.D. 1367/2007 (19), other conditions being equal, we proceed to correct the noise level inside the cabin when the incubator is on with the levels existing when it is off. From these calculations, it is obtained that the Giraffe incubator provides a noise level of 53 dBA, while the OCP 3000 transmits 64 dBA, due to the presence of emerging low frequency and tonal components.

If the existing noise levels inside the incubator are correlated with those present in the NICU (during 24 h), it is verified that the variations inside the incubator are very small between the day and night periods, remaining very stable and with values higher than those recommended at all times (9). This fact indicates that the noise inside the incubator cabin is hardly influenced by the noise generated in the NICU since its background noise is very high.

### Regarding the source of noise

As has been shown, the motor that drives the fan capable of producing the ideal conditions inside the cabin is the main source of noise perceived by the newborn. The main hypothesis that arises, due to the existing needs is the elimination of it, which would lead to the elimination of the structural noise and vibrations generated by the current fans incorporated in the incubator, being replaced by an external system that allows a significant improvement in the supply of air and oxygen flow while ensuring a certain level of constant (and adjustable) pressurization inside the neonatal cabin.

Another possibility would be to move the engine as far as possible from the cabin. For the transport of airflow, from the engine to the neonatal cabin, both elements should be connected through flexible ducts, which although it would be necessary to study, as a general rule, the sound produced by the engine could be transmitted and even increased due to this duct. The solution is to attach filters, mainly reactive silencers since they are of great importance in noise control.

### Regarding the neonate compartment

It is possible to intervene acoustically in two different and complementary ways in the incubator cabin: (i) through the selection of materials and (ii) through the design of the shape of the cover. In the first case, as has been shown previously, the incorporation of sound-absorbing material inside the compartment has not meant a great deal of noise attenuation and it also has the problem of being a porous material and, therefore, can harbor bacteria and microorganisms. Therefore, the most feasible option would be to create a double cover with independent lids. The outer cover would not be different from the current ones, but with a careful design of the forms and maintaining the aforementioned medical advantages. The inner cover would use absorbent materials creating a selective absorbent.

**FIGURE 6:**
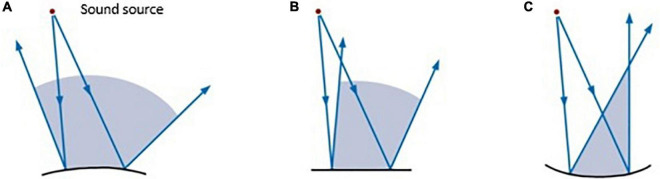
Coverage areas are associated with different reflective surfaces: **(A)** convex surface; **(B)** flat surface; **(C)** concave surface (20).

In the absence of a study on the reverberation time existing in the incubator, it is logical to think that since it is made of reflective materials, it is high, so the shape of the dome of the cabin plays a significant role in this matter. The aim would be to remove as far as possible the first reflections of the newborn’s auditory system. Figure 6 shows the behavior offered by different shapes when sound is reflected on their surface.

## Conclusion

**FIGURE 7:**
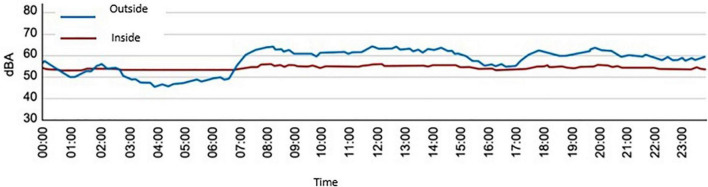
Sound pressure levels (LAeq) over 24 h, measured inside and outside an incubator located in the NICU of the Puerta del Mar University Hospital, in Cádiz.

Given the results obtained, if the ambient noise in the room is less than the highest level produced by the incubator motor, the noise generated by the motor inside the cabinet will dominate, with levels of approximately 56 dBA. Likewise, simply starting the engine can cause an increase, in the worst of the analyzed cases, of up to 22 dBA inside neonatal cabin and 3 dBA outside (shown in Figure 7).

However, the incubator motor not only produces this increase in the sound pressure level but also produces, depending on the studied model, a greater or lesser acoustic effect on the neonate, reaching an LKeq, T Source (associated with annoyance) of up to 64 dBA, due to the presence of emerging and low-frequency tonal components.

When the ambient noise in the room is much higher than that generated by the motor, the walls that make up the incubator compartment produce an attenuation of up to 10 dBA. The attenuation will be maximum when the difference between the noise level inside and outside is at least 10 dBA. The attenuation will be minimal when the difference between the outside level and the inside level is zero.

The noise inside the incubator cabin is hardly influenced by the noise generated in the NICU since its background noise is very high.

A solution to eliminate the noise of the incubator is the elimination of the motor/fan system by another type of system, which would mean the elimination of structural noise and vibrations.

## Data availability statement

The original contributions presented in this study are included in the article/supplementary material, further inquiries can be directed to the corresponding author.

## Author contributions

RH-M wrote first draft of the manuscript. All authors contributed to the study conception and design, performed material preparation and data collection and analysis, commented on previous versions of the manuscript, read, and approved the final manuscript.
